# Integrated DNA methylation and gene expression analysis in the pathogenesis of coronary artery disease

**DOI:** 10.18632/aging.101847

**Published:** 2019-03-07

**Authors:** Liu Miao, Rui-Xing Yin, Qing-Hui Zhang, Xi-Jiang Hu, Feng Huang, Wu-Xian Chen, Xiao-Li Cao, Jin-Zhen Wu

**Affiliations:** 1 Department of Cardiology, Institute of Cardiovascular Diseases, the First Affiliated Hospital, Guangxi Medical University, Nanning 530021, Guangxi, China; 2 Guangxi Key Laboratory Base of Precision Medicine in Cardio-cerebrovascular Disease Control and Prevention, Nanning 530021, Guangxi, China; 3 Guangxi Clinical Research Center for Cardio-cerebrovascular Diseases, Nanning 530021, Guangxi, China; 4 Department of Neurology, the First Affiliated Hospital, Guangxi Medical University, Nanning 530021, Guangxi, China

**Keywords:** coronary artery disease, function enrichment, DNA methylation-mRNA expression-CAD interaction network, correlation analyses

## Abstract

To evaluate DNA methylation sites and gene expression associated with coronary artery disease (CAD) and the possible pathological mechanism involved, we performed (1) genome-wide DNA methylation and mRNA expression profiling in peripheral blood datasets from the Gene Expression Omnibus repository of CAD samples and controls; (2) functional enrichment analysis and differential methylation gene regulatory network construction; (3) validation tests of 11 differential methylation positions of interest and the corresponding gene expression; and (4) correlation analysis for DNA methylation and mRNA expression data. A total of 669 differentially expressed mRNAs were matched to differentially methylated genes. After disease ontology, Kyoto Encyclopedia of Genes and Genomes pathway, gene ontology, protein-protein interaction and network construction and module analyses, 11 differentially methylated positions (DMPs) corresponding to 11 unique genes were observed: *BDNF* – cg26949694, *BTRC* - cg24381155, *CDH5* - cg02223351, *CXCL12* - cg11267527, *EGFR* - cg27637738, *IL-6* - cg13104385, *ITGB1* - cg20545410, *PDGFRB* - cg25613180, *PIK3R1*- cg00559992, *PLCB1* - cg27178677 and *PTPRC* - cg09247619. After validation tests of 11 DMPs of interest and the corresponding gene expression, we found that *CXCL12* was less hypomethylated in the CAD group, whereas the relative expression of *ITGB1*, *PDGFRB* and *PIK3R1* was lower in CAD samples, and *CXCL12* and *ITGB1* methylation was negatively correlated with their expression. This study identified the correlation between DNA methylation and gene expression and highlighted the importance of *CXCL12* in CAD pathogenesis.

## Introduction

Coronary artery disease (CAD) remains one of the most common causes of death worldwide. As a complex and multifactorial disorder, it introduces a heavy economic and social burden to people of all countries worldwide [1]. Atherosclerosis is the common pathological basis for CAD and other related diseases such as myocardial infarction (MI), peripheral artery disease and stroke [2]. Numerous epigenetic factors and their interactions can contribute to CAD, such as chromatin remodeling, DNA methylation, noncoding RNA regulation and histone modification [3]. Among these factors, DNA methylation is a key epigenetic process for atherosclerosis and CAD [4].

Several previous studies have examined relationship between CAD development and DNA methylation. When the DNA methylation of critical genes is changed, CAD occurs [5]. DNA methylation can also inhibit the hub genes from functioning in CAD. Following gene promoter hypermethylation, gene expression will be downregulated. Conversely, when DNA is hypomethylated, the expression level of the gene will be upregulated.

To identify more novel CAD-associated DNA methylation sites, we carried out and integrated two microarray datasets from the Gene Expression Omnibus (GEO) repository (methylome and transcriptome), and we constructed an integrative regulatory network of CAD-related differential methylation and matched differential expression of genes (DMaGs). Subsequently, we also validated several DMaGs in an additional sample to evaluate the potential DNA methylation-mRNA expression-CAD regulatory effect.

## RESULTS

### Data preprocessing and identified differentially methylated positions (DMPs) and differentially expressed genes (DEGs)

**Figure 1:**
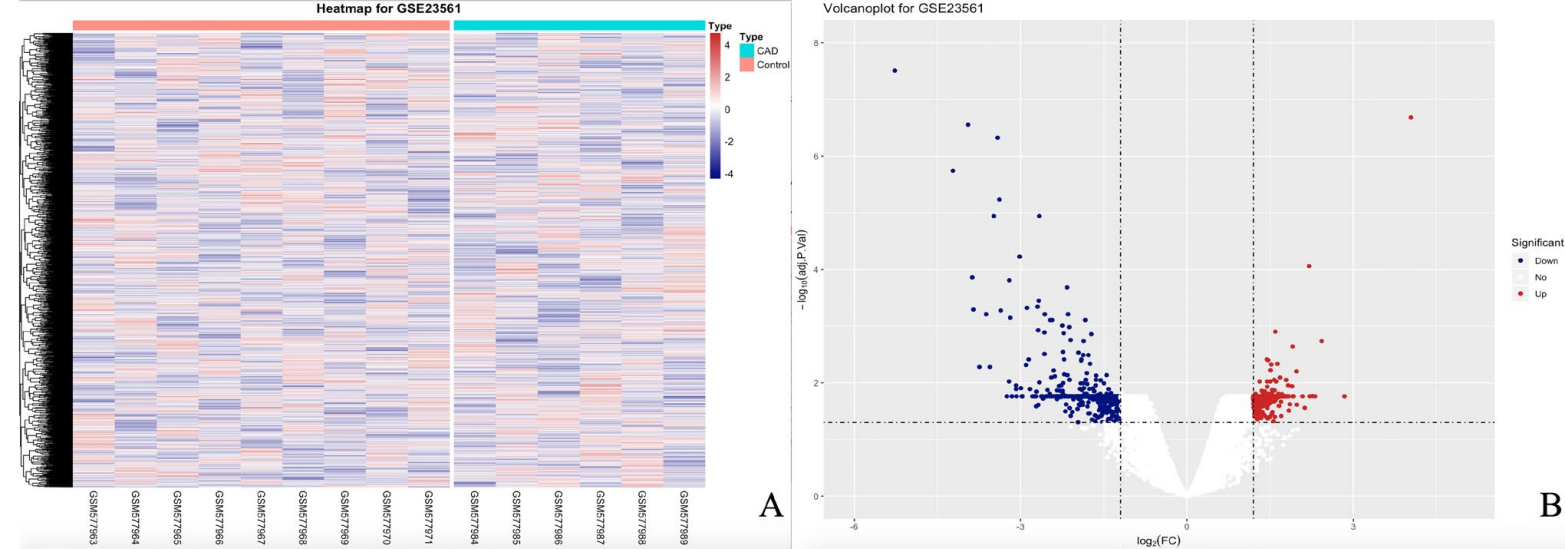
**The heatmap and volcanoplot for DEGs.** (**A**) For the heatmap, the control samples are shown as a red cluster, and the CAD samples are shown as a green cluster. (**B**) For the volcano plot, the two vertical lines show the 1.2-fold change boundaries and the horizontal line the statistical significance boundary (adj-*P* < 0.05). Items with statistical significance and upregulation are marked with red dots, and downregulated with blue dots in the volcano plot.

When each gene expression and profile was analyzed from GSE23561, we obtained a total of 54 560 expression probes. After the data were analyzed, 3 882 DEGs were obtained, of which 471 were downregulated, and 3 411 upregulated. The heat map and volcano plot of the DEGs are presented in Fig. 1.

**Figure 2:**
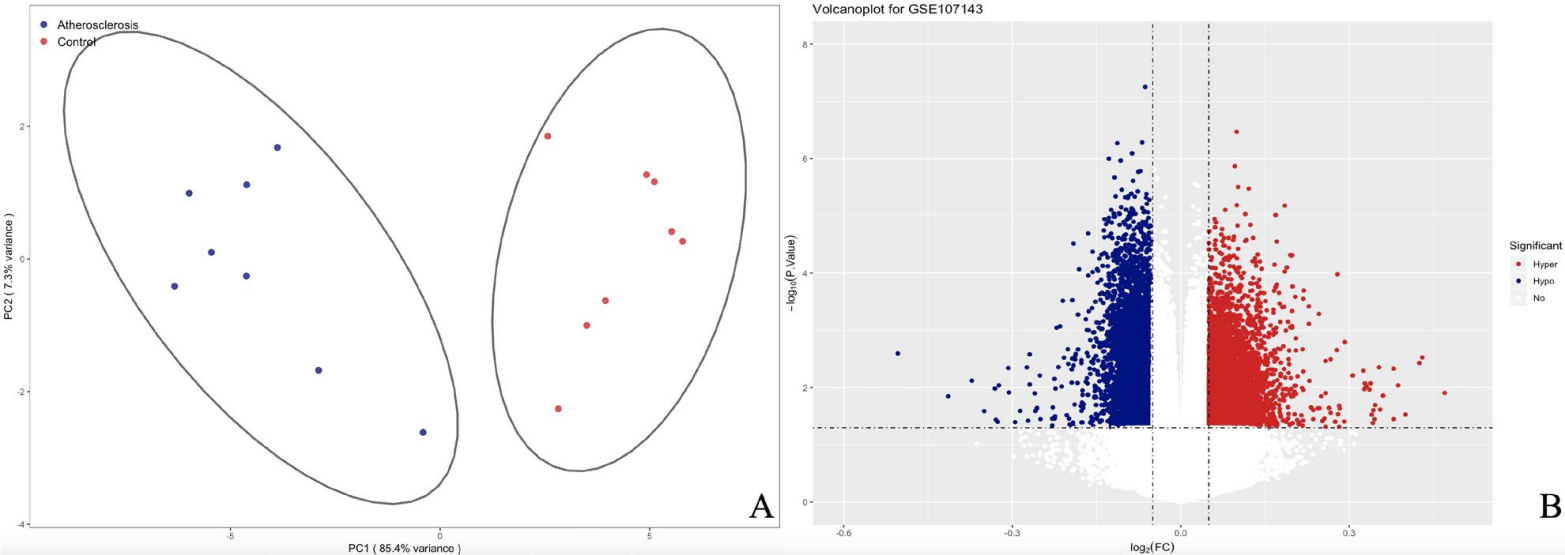
**The PCA and volcanoplot for DMPs.** (**A**) For the PCA map, the red strip represents the healthy control and the blue strip atherosclerosis samples. (**B**) For the valcano plot, the two vertical lines are the 0.05-fold change boundaries and the horizontal line is the statistical significance boundary (*P* < 0.05). Items with statistical significance and hypermethylation are marked with red dots and hypomethylation are marked with blue dots in the volcano plot.

We measured DNA methylation levels at 460 295 methylation sites in GSE107143. After quality control and screening procedure, 454 325 methylation positions were subjected to differential analysis. In total, 1 2559 DMPs, including 5 015 hypermethylated and 7 544 hypomethylated DMPs were identified. According to the annotation, 8707 DMPs were physically located within 4558 unique genes. The principal component analysis (PCA) map and volcano plot of DEGs are presented in Fig. 2.

**Figure 3:**
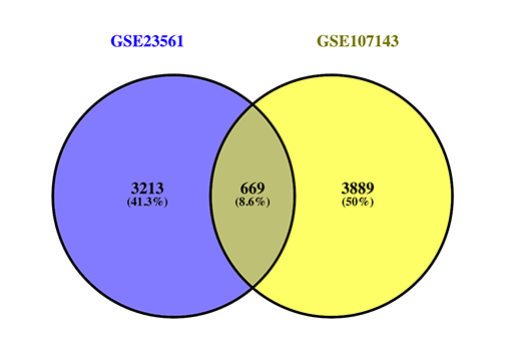
Venn map showing the intersection of DEGs and DMGs.

When differentially methylated genes (DMGs) were matched to the DEGs, approximately 669 genes (DMaGs) had been selected for subsequent analysis (Figure 3). The details of the 669 genes are shown in Supplementary Table 1.

### Analysis of DMaG functional enrichment

We performed functional and pathway enrichment analysis to identify genes with the same function and pathway in CAD for further analysis. In the analysis of gene ontology (GO) functions, approximately 150 biological processes, 45 cellular components, and 7 molecular functions were identified with an adjusted *P* < 0.05. Approximately 50 pathways were enriched for the Kyoto Encyclopedia of Genes and genomes (KEGG) pathway and 115 disease ontology (DO) item analysis of the screened DMGs at *P* < 0.05 (false discovery rate, FDR set at < 0.2).

Among these items, GO:0007156 homophilic cell adhesion via plasma membrane adhesion molecules, GO:0098742 cell-cell adhesion via plasma-membrane adhesion molecules, GO:0016339 calcium-dependent cell-cell adhesion via plasma membrane cell adhesion molecules, GO:0050808 synapse organization, GO:0061564 axon development, GO:0043235 receptor complex, GO:0000982 transcription factor activity, RNA polymerase II proximal promoter sequence-specific DNA binding and GO:0045296 cadherin binding in GO functions; hsa04750 inflammatory mediator regulation of transient receptor potential (TRP) channels, hsa04020 calcium signaling pathway, hsa04060 cytokine-cytokine receptor interaction, hsa04514 cell adhesion molecules (CAMs), hsa04010 mitogen-activated protein kinase (MAPK) signaling pathway and hsa04151 phosphatidylinositol 3' -kinase-Akt (PI3K-Akt) signaling pathway in the KEGG pathways; DOID:423 myopathy, DOID:9352 type 2 diabetes mellitus, DOID:5844 myocardial infarction, DOID:0050700 cardiomyopathy, DOID:3393 coronary artery disease and DOID:9970 obesity in Disease Ontology were related to CAD. The genes related to these items were selected for further analysis.

**Figure 4:**
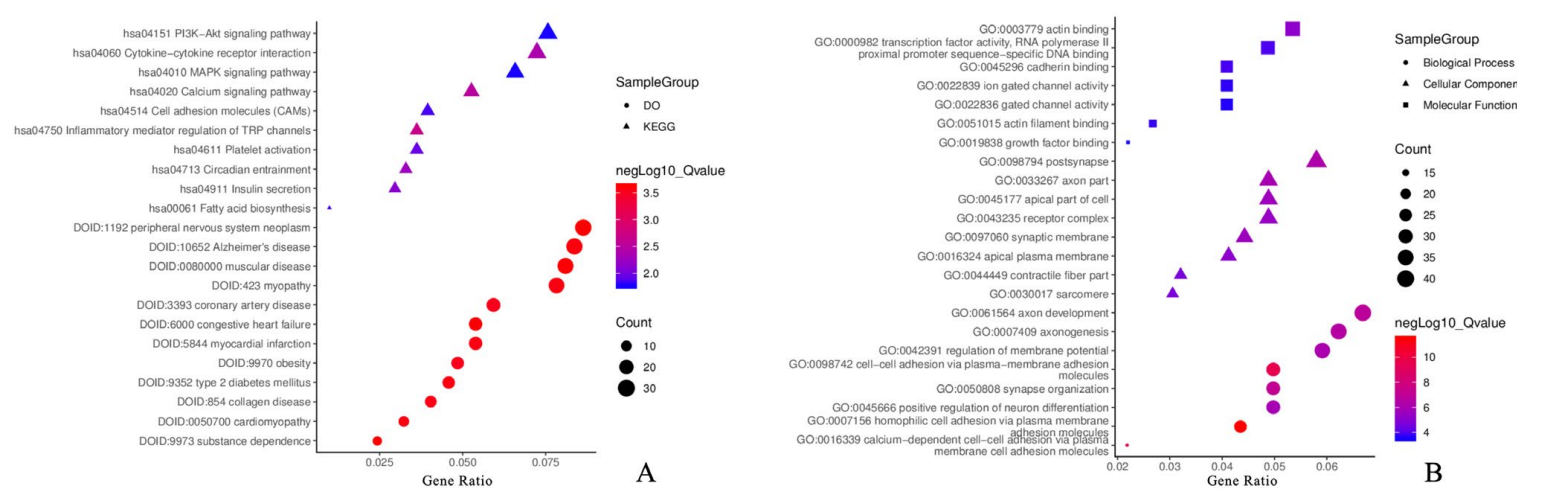
**Functional annotation for DMaGs.** (**A**) KEGG and DO analysis for DMaGs; (**B**): GO analysis for DMaGs.

Figure 4 represents the most valuable items in the development of CAD; detailed information is provided in Supplementary Table 2.

### Protein-protein interaction (PPI) network construction and submodule analysis

**Figure 5:**
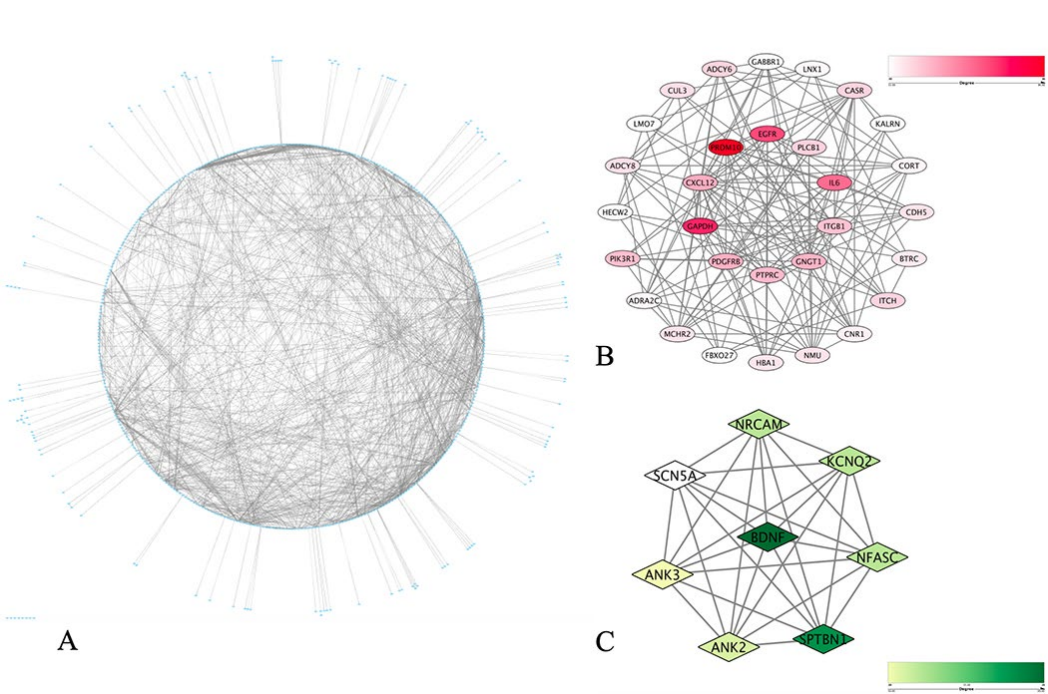
**PPI network construction and identification of hub items.** (**A**) PPI network of the selected DMaGs. The edge shows the interaction between two genes. Significant modules identified from the PPI network using the molecular complex detection method with a score > 6.0. (**B**) Molecular-1 with MCODE = 11; (**C**) Molecular-2 with MCODE = 8. A degree was used to describe the importance of protein nodes in the network, with a dark color filling denoting a high degree and light color a low degree.

To elucidate the PPI in these matched genes, data analysis was performed using the STRING database, from which 1 662 protein pairs and 397 nodes were revealed with a combined score > 0.9. Fig. 5A shows the net analysis in Cytoscape. For detection using the Molecular Complex Detection (MCODE) app, two modules with a score > 6 were found and are presented in Fig. 5B and Fig. 5C. These two modules included a total of 38 genes. After a comprehensive analysis of the GO, DO, and KEGG data, we selected 11 DMaGs related to the onset of CAD, which demonstrated a high degree of association simultaneously, as well as in the submodule analysis. These genes included brain-derived neurotrophic factor (*BDNF*), beta-transducing repeat containing E3 ubiquitin protein ligase (*BTRC*), cadherin 5 (*CDH5*), C-X-C motif chemokine ligand 12 (*CXCL12*), epidermal growth factor receptor (*EGFR*), interleukin-6 (*IL-6*), integrin subunit beta 1 (*ITGB1*), platelet derived growth factor receptor beta (*PDGFRB*), phosphoinositide-3-kinase regulatory subunit 1 (*PIK3R1*), phospholipase C beta 1 (*PLCB1*) and protein tyrosine phosphatase receptor type C (*PTPRC*), and the details are presented in Table 1.

**Table 1:** The matched pairs of DEGs and DMPs.

DMPs						DEGs	
SYMBOL	CpG site	MAPINFO	CHR	Δβ	*P* values	Fold Change	*P* values
BDNF	cg26949694	27740059	11	9.09E-02	1.13E-02	1.317611	1.77E-03
BTRC	cg24381155	101351808	10	-6.02E-02	2.04E-03	1.2535658	4.28E-03
CDH5	cg02223351	55017021	7	-6.66E-02	0.00589	1.2556628	3.35E-03
CXCL12	cg11267527	44290098	10	-7.92E-02	9.84E-03	1.4688135	1.91E-03
EGFR	cg27637738	66364607	16	-7.02E-02	5.63E-03	1.3553032	6.61E-04
IL6	cg13104385	22723889	7	9.79E-02	2.97E-02	1.2966267	1.80E-03
ITGB1	cg20545410	32898318	10	-5.96E-02	2.04E-03	1.2151364	1.04E-03
PDGFRB	cg25613180	149510300	5	-5.80E-02	1.69E-02	1.252214	2.28E-03
PIK3R1	cg00559992	67582213	5	6.07E-02	1.97E-02	1.367476	8.36E-04
PLCB1	cg27178677	8130265	20	-7.14E-02	2.36E-02	1.2294703	3.35E-03
PTPRC	cg09247619	198636968	1	-7.38E-02	1.94E-04	1.4577283	2.75E-02

CHR: chromosome. DEG: differentially expressed gene. Δβ: difference of methylation between patients with CAD and healthy controls. DMP: differential methylation position. MAPINFO: position in Build 37.

### Validation of the DNA methylation–mRNA regulatory net

**Figure 6:**
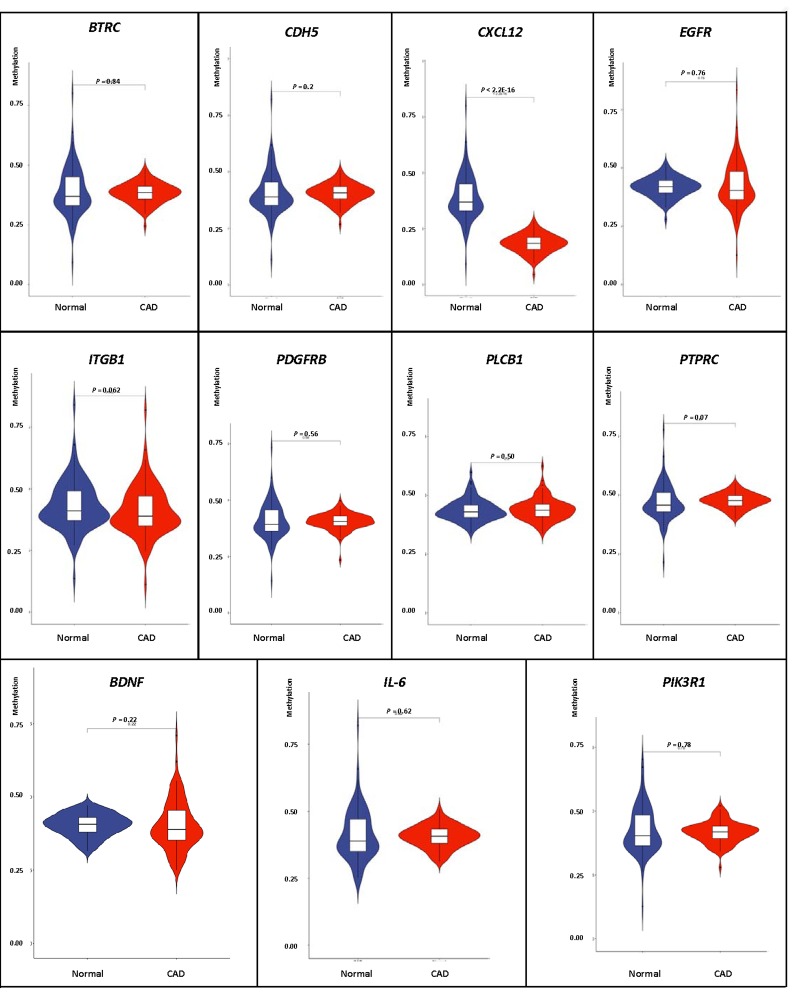
Verification of DNA methylation of interest between CAD and healthy samples.

**Figure 7:**
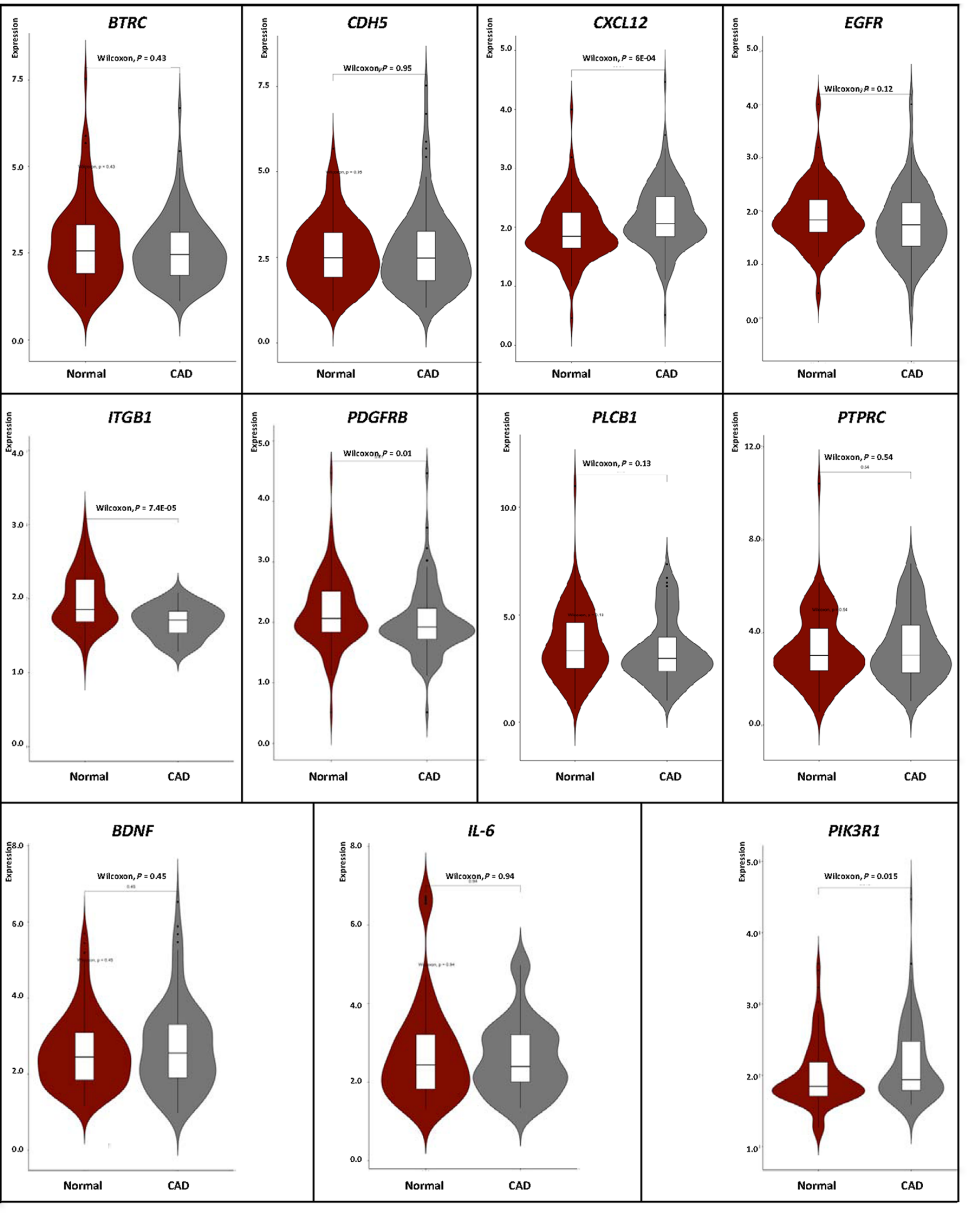
Verification of mRNA expression of interest between CAD and healthy samples.

**Figure 8:**
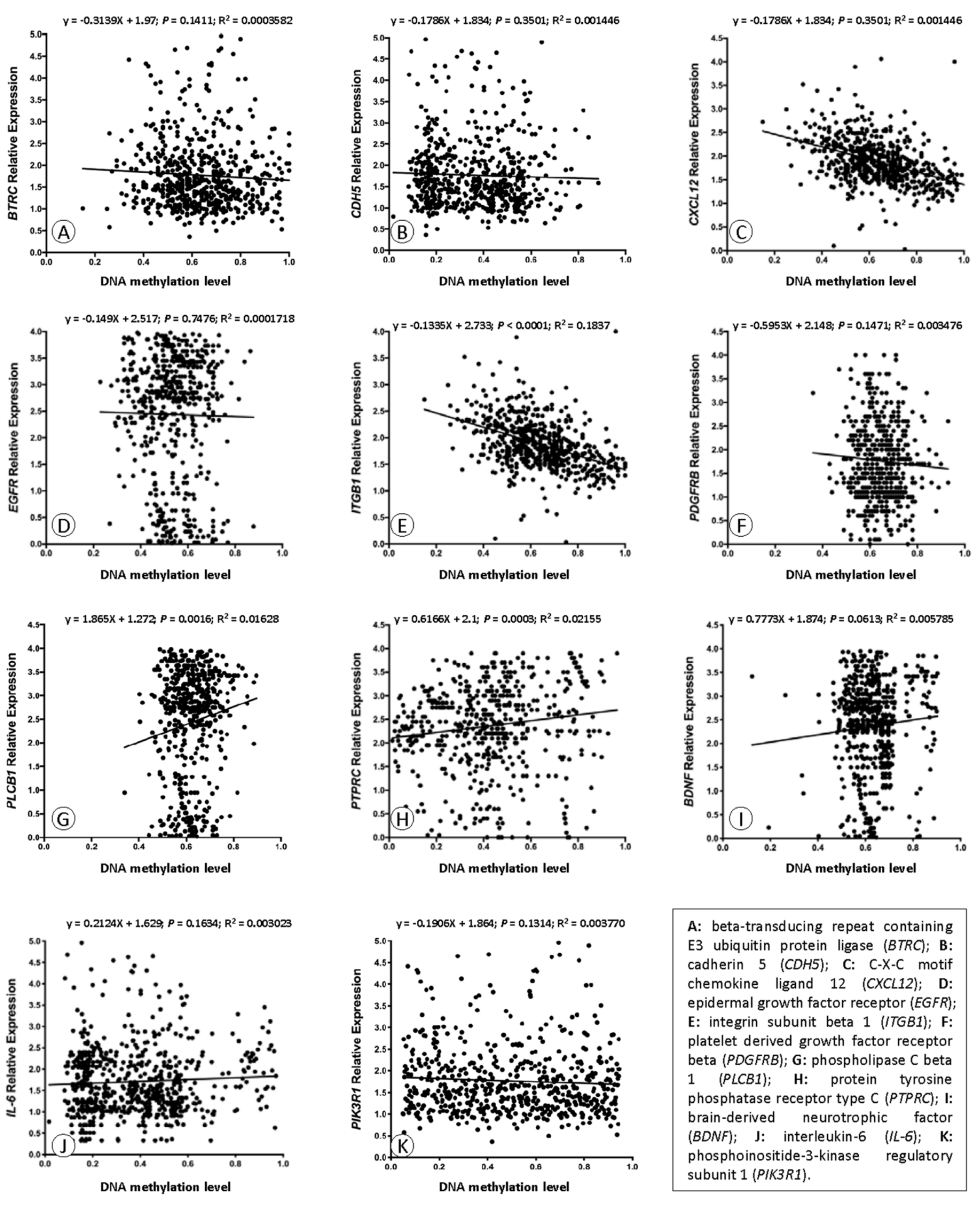
Correlation analyses of DNA methylation and mRNA expression.

We carried out correlation analysis to detect whether DNA methylation causes CAD by regulating gene expression. The addition of methyl groups at cytosine-guanine dinucleotides (CpGs) in regulatory/promoter regions in DNA, known as DNA methylation, typically leads to transcriptional repression and decreased expression of the gene in question [6]. However, the DNA methylation was positively correlated with the gene expression in *BDNF, IL-6* and *PIK3R1.* We selected all the methylation-mRNA pairs to evaluate the relationship between DNA methylation and gene expression in a total of 606 samples (303 healthy controls and 303 CAD patients). The 606 verification samples were matched for age and gender. The weight, body mass index (BMI), waist circumference, smoking status, serum glucose, serum total cholesterol (TC) and low-density lipoprotein cholesterol (LDL-C) levels were higher in the CAD than in the control groups (Table 2). Eleven DMPs corresponding to 11 unique genes were identified, including *BDNF* – cg26949694, *BTRC* - cg24381155, *CDH5* - cg02223351, *CXCL12* - cg11267527, *EGFR* - cg27637738, *IL-6* - cg13104385, *ITGB1* - cg20545410, *PDGFRB* - cg25613180, *PIK3R1*- cg00559992, *PLCB1* - cg27178677 and *PTPRC* - cg09247619. First, we analyzed the methylation of these 11 genes in two samples, and we found that *CXCL12* was less hypomethylated in the CAD group (Fig. 6). Then, the relative expression of *CXCL12* was found to be higher in CAD samples, whereas those of *ITGB1*, *PDGFRB* and *PIK3R1* were lower in CAD samples (Fig. 7). Next, we performed a correlation analysis between DNA methylation and gene expression in the same samples, and we found that *CXCL12* and *ITGB1* methylation was negatively correlated with their respective expression (Fig. 8). After a comprehensive analysis, we found that *CXCL12* DNA methylation had a significantly negative correlation with its expression.

**Table 2:** Comparison of the demographic, lifestyle characteristics and serum lipid levels between the normal and CAD groups.

Parameter	Control	CAD	*test-statistic*	*P*
Number	303	303		
Male/female	92/211	96/207	0.675	0.409
Age (years)^1^	56.31±10.45	56.88±10.23	0.944	0.383
Height (cm)	155.23±6.92	155.58±7.12	1.594	0.222
Weight (kg)	51.86±7.54	60.56±11.82	21.439	1.77E-005
Body mass index (kg/m^2^)	28.49±3.23	31.30±6.25	27.214	2.11E-008
Waist circumference (cm)	73.43±6.61	87.45±9.87	23.122	3.34E-005
Smoking status [*n* (%)]	78(26.0)	105(34.8)	7.690	0.005
Alcohol consumption [*n* (%)]	72(23.9)	77(25.5)	0.309	0.578
Systolic blood pressure (mmHg)	128.23±17.28	129.57±25.16	0.513	0.442
Diastolic blood pressure (mmHg)	80.54±10.16	82.49±13.35	0.717	0.291
Pulse pressure (mmHg)	49.64±14.13	50.42±14.59	1.492	0.233
Glucose (mmol/L)	5.91±1.76	7.64±2.73	17.867	5.02E-005
Total cholesterol (mmol/L)	4.93±1.13	5.34±1.16	7.131	0.016
Triglyceride (mmol/L)^2^	1.49(0.51)	1.53(1.22)	2.137	0.187
HDL-C (mmol/L)	1.52±0.44	1.06±0.26	8.673	0.013
LDL-C (mmol/L)	2.84±0.84	2.88±0.79	9.497	0.007
ApoA1 (g/L)	1.23±0.25	1.17±0.27	0.384	0.518
ApoB (g/L)	0.83±0.19	0.89±0.32	1.542	0.193
ApoA1/ApoB	1.67±0.50	1.66±0.57	0.095	0.758

HDL-C: high-density lipoprotein cholesterol. LDL-C: low-density lipoprotein cholesterol. Apo: Apolipoprotein. ^1^ Mean ± SD determined by the *t*-test. ^2^ Because the data were not normally distributed, the value obtained for triglyceride was presented as the median (interquartile range), and the difference between the two groups was determined using the Wilcoxon-Mann-Whitney test.

## DISCUSSION

An increasing number of studies have approved the relationship between DNA methylation and CAD development over the past two decades. In general, the relationship between methylation and genes is mainly reflected in the following two aspects. When hypermethylation occurs in the genes promoter region, the transcription process is inhibited, resulting in downregulation of gene expression. When the promoter region is hypomethylated, the opposite effect is observed. Another strategy is to observe the change in the phenotype after application of a DNA methylation inhibitor. *In vitro,* the key gene in CAD is typically targeted, and then methylation of the gene or gene promoter is detected [7]. In human studies, genome-wide methylation has been carried out comparing healthy control and CAD samples with HumanMethylation array or sequencing [8]. Subsequently, the methylation of key genes was also validated in human tissue by pyrosequencing or methylationspecific PCR (MSP) [9]. In the present study, we integrated two sets of gene microarray data (DNA methylation and gene expression) for peripheral blood samples from arteriosclerosis patients. These two datasets had matching age structures, arteriosclerosis diseases and source of microarray samples sources. We identified genes that with the same differential methylation and differential expression. Finally, in CAD and normal samples, we validated that *CXCL12* cg11267527 methylation was related to the development of CAD.

Recently, several studies have demonstrated that elevated serum *CXCL12* expression is positively correlated with the incidence of CAD [10,11]. Chemokines, for the sake of similarities in their amino acid sequences, can be defined on a structural basis into four major families, including the CXC, CX3C, CC and C subclasses [12]. Previous studies have shown that chemokine CXC classes, such as *CXCL12*, played important role in the process of angiogenesis and angiostasis [13]. According to the presence or absence of an ELR (glutamine-leucine-arginine) motif immediately adjacent to CXC, the CXC chemokines are further divided in two subgroups, ELR+ and ELR−. The ELR− subgroup of CXC chemokines, such as *CXCL12* is a chemoattractant for lymphocytes, monocytes and NK cells [14]. Genome-wide association studies (GWASes) in more than 100 000 people have revealed novel loci associated with CAD and MI, presenting exciting opportunities to discover novel disease pathways. One of the identified loci is on chromosome 10q11, near the gene for the chemokine *CXCL12,* which has been implicated in CAD in both mouse and human studies. These GWASes demonstrate that CXCL12 may emerge as a potential therapeutic target for atherosclerosis and thrombosis [15]. All these causes can be attributed to the inflammatory process. Several previous studies have shown that atherosclerosis is a chronic inflammatory reaction, comprising two remarkable steps: smooth muscle cell recruitment and foam cell formation. *CXCL12* can also play a crucial role in the accumulation of smooth muscle progenitor cells (SPCs), participate in the inflammatory response and induce endothelial cells differentiation into foam cells, eventually lead to arteriosclerosis [16]. In the current research, we found that the *CXCL12* cg11267527 was in the promoter region of the gene. In this case, combined with previous studies, we speculated that hypomethylation of the promoter region might lead to the upregulation of gene expression, which was increased during the inflammatory response, resulting in endothelial cells differentiation into foam cells to cause vascular endothelial injury and eventually atherosclerosis.

Additionally, smoking is a risk factor for many human diseases, and DNA methylation has been related to smoking [17]. Zhu et al. have identified DNA methylation markers associated with smoking in a Chinese population, including some markers that have also been correlated with gene expression. Exposure to naphthalene, a byproduct of tobacco smoke, may contribute to smoking-related methylation [18]. Zhang et al. found that smoking is closely related to DNA methylation and increases all-cause mortality in cardiovascular diseases [19]. In our study, we found that the percentage of smoking in CAD patients was higher than in controls. One reasonable explanation for this observation is that smoking caused changes in DNA methylation, resulting in abnormal gene expression and leading to the onset of the disease.

The present study has some limitations. First, although the microarray samples had a matched age structure, as well as the same diseases and sample resources, the batch effect would still lead to some deviation in the final results. Second, the sample size of the microarray was somewhat small. Although our study has analyzed two different types of datasets, a low statistical efficiency was still unavoidable. Third, the patients in this study were from one hospital. Thus, it is unknown whether there was a difference would be observed for patients from different areas and of various races. Therefore, the validity of this analysis should be further tested in more prospective cohorts. Finally, the specific mechanism of the (DNA methylation)-mediator (mRNA)-outcome (CAD) net for regulating the pathogenesis of CAD has not been fully validated *in vivo* and *in vitro*.

In conclusion, we downloaded 2 datasets from GEO and combined differentially methylated and expressed genes. After functional analysis, we selected 11 DMaGs for validation in 606 samples (303 CAD patients and 303 healthy controls). *CXCL12* was found to be hypomethylated and to exhibit upregulated gene expression in CAD patients. Additionally, correlation analysis showed that DNA methylation caused CAD by regulating gene expression.

## MATERIALS AND METHODS

### Microarray data

Two profile data sets were selected. GSE23561 [20] was retrieved from the GPL10775 Human 50K Exonic Evidence-Based Oligonucleotide array for gene expression. Microarrays were performed for 35 subjects, including 6 CAD patients and 9 healthy controls (mean age = 50.53 ± 12.17, 7 males and 8 females). Log (base 2) transformation of the downloaded raw data was performed with the GEOquery module available in R [21]. The CEL files were transformed into the expression value matrix using the Affy package in R with robust multiarray (RMA) methods to normalize the expression value matrix [22]. Subsequently, Bioconductor in R was used to convert the probe data to the gene [23]. The expression of some probes was too high or too low. We determined that such probes were outliers and had to be removed without further analysis. In cases in which many probes corresponded to one gene, we used the average expression value to screen for differential genes. For any gene that corresponded to multiple probes, the mean expression value of such a gene should be chosen. GSE107143 [24] was designed as bisulfite converted DNA from whole the blood of 8 atherosclerotic patients and 8 healthy subjects were hybridized to the Illumina Infinium HumanMethylation 450 BeadChip (mean age = 62.25 ± 18.18, 16 males and no females). These data were processed using the GEO2R online analysis tool (https://www.ncbi.nlm.nih.gov/geo/geo2r/) to compare atherosclerotic patient samples and normal samples [25]. The samples for these two datasets were age-matched (*P* = 0.062), and the source of the microarrays was peripheral blood.

### DEGs and DMGs analysis

The limma package in R [26] enabled the identification of DEGs based on the comparisons between the control and the CAD samples, and provided good normalization of the expression matrix. The threshold values were |log_2_-fold-change| > 1.2 and *P* < 0.05 (FDR < 0.2). The DMPs were calculated by GEO2R based on comparisons between the controls and atherosclerotic patients. DMPs located in the gene region were assigned to the corresponding genes, which were defined as DMGs. The threshold values were | log_2_-fold-change | (Δβ) > 0.05 and *P* < 0.05 (FDR < 0.2). Subsequently, the differentially methylated genes were matched to the DEGs, and only the matched genes (DMaGs) could be selected for further analysis.

### DMaGs functional enrichment analysis

Studies of large-scale transcription data or genomic data are usually performed based on functional enrichment analyses consisting of DO, KEGG pathway and GO analyses. In the current study, we used clusterProfiler and DOSE package in R [27] to complete the above analysis.

### PPI network construction and module analysis

The protein prediction and experimental interactions were analyzed using the STRING database (version 10.5) [28]. Gene fusion, coexpression experiments, databases, text mining, neighborhoods and cooccurrence are typical prediction methods for the database. In addition, a combined fraction was used to show the interaction of protein pairs in the database. In this study, DMaGs were mapped to PPIs, and a combined score > 0.9 was used as the cutoff value [29] to analyze key genes in the network. Degrees served as a valuable way to determine the role of protein nodes in the network. Network modules represented one of the cores of the protein networks and may have specific biological implications. The MCODE in the Cytoscape software package (version 3.61) [30,31] was used to identify the major and the most notable clustering modules. Subsequently, we choose EASE ≤ 0.05 and a count ≥ 2 for the cutoff value, and a MCODE score > 6 as the threshold for further analysis.

### Sample verification and diagnostic criteria

A total of 606 subjects had complaints about their chests in the First Affiliated Hospital, Guangxi Medical University from Jan. 1, 2015 to Dec. 31, 2017. The levels of TC, triglyceride (TG), high-density lipoprotein cholesterol (HDL-C), LDL-C, apolipoprotein (Apo) A1, ApoB and the ratio of ApoA1 to ApoB defined as normal values were 3.10–5.17, 0.56–1.70, 0.91–1.81, 2.70–3.20 mmol/L, 1.00–1.78, 0.63–1.14 g/L, and 1.00–2.50; respectively [32]. Since angiographic examination was performed for CAD and/or other suspected diseases, all participants were examined using coronary angiography by two experts. CAD was confirmed as more than one of the three major coronary arteries or their major branches (branched diameter ≥ 2 mm) (≥ 50%) [33]. Participants who had a history of CAD, type I diabetes mellitus and congenital heart disease were excluded. Through clinical examination, medical history and questionnaires, the control group was judged to have no CAD. Medical history and general information were obtained using a standard questionnaire [34]. The investigation conformed to the rules of the Helsinki Declaration of 1975 (http://www.wma.net/en/30publications/10policies/b3/) and the new edition of 2008. The research design was approved by the Ethics Committee of the First Affiliated Hospital of Guangxi Medical University (No: Lunshen-2011-KY-Guoji-001; March 7, 2011). All procedures are conducted in conformity to ethical standards. Written informed consent was obtained from all the participants upon receipt of a complete explanation of the study. In the initial evaluation, all clinical data were collected based on the medical records. Clinical data collection and biochemical measurements were performed according to previous studies [35].

### Validation of differential methylation and mRNA expression of DMaGs of interest

Focused on identified regulatory network, we selected eight DMaGs to validate methylation and gene expression in an additional sample, respectively. These selected DMaGs met the following criteria: (1) the DMP was located in the promoter region; (2) the methylation level of the DMP was significantly correlated with gene expression; (3) the CpG dinucleotide content was enriched in the 300-bp up- and downstream regions around the DMP; and (4) genes in the identified regulatory network were prioritized. Targeted bisulfite sequencing was used to validate the DMPs. Primer design and optimization were performed by GeneSky Corporation (Shanghai, China). The primers were designed to flank each targeted CpG site and span 100-300 bp. The primers information is summarized in the Supplementary Table 3.

Peripheral blood samples were obtained from CAD patients and healthy controls. The peripheral blood mononuclear cells (PBMCs) were isolated from 15 ml peripheral blood by density gradient centrifugation using Lymphoprep (Sigma, life science, USA) within 4 hours after phlebotomy. Total RNA was isolated from PBMCs by standard phenol-chloroform extraction using TRIzol reagent (Invitrogen Life Technologies, Carlsbad, USA) according to the manufacturer’s instructions, and the concentration was measured using a Nanodrop ND-1000 Spectrophotometers (Thermo Fisher Scientific, Waltham, USA). The RNA quality was checked using a Bioanalyzer Nanochip (Agilent Technologies, Santa Clara, USA). Genomic DNA was isolated using genomic DNA extraction kits (Life Technologies, Gaithersburg, USA), and the DNA integrity was analyzed by agarose gel electrophoresis. Following primer validation, study specimen DNA was bisulfite-converted using the EZ DNA Methylation-Gold Kit (ZYMO, CA, USA). Samples were amplified, barcoded and sequenced (MiSeq, Illumina, Inc., San Diego, USA) using the paired-end sequencing protocol according to the manufacturer’s guidelines.

Quantitative real-time PCR (qRT-PCR) was used to validate the differential expression for the above eleven selected DMaGs. Total RNA from PBMCs was isolated using TRIzol reagent, according to the instructions recommended by the manufacture, and reverse-transcribed. Reverse transcription was performed in a total volume of 20 μl, which contained 1 μl oligo (dT) (100 μM), RNase-free water 11 μl, 25 mM each dNTPs 0.4 μl, recombinant RNase inhibitor 0.5 μl, Moloney murine leukemia virus (M-MLV) 0.5 μl, 5 × M-MLV buffer 4 μl and RNase-free water 2.6 μl, and took place under the conditions of 42 °C for 60 min, 85 °C for 5 min, and 4 °C forever. The cDNA served as template for amplification by qPCR using SYBR Green assays. The assay was performed using a SYBR Green MasterMix kit in a volume of 10 μl, which contained 0.2 × cDNA 1 μl, 2 × SYBR master mix 5 μl and 0.625 mM each primer 4 μl. The cDNA amplification was monitored using a Roche light cycler 480II under 1 cycle of pre-degeneration of 95°C for 10 min, 40 cycles of 95 °C for 10 s and 60 °C for 20 s. This assay was carried out in triplicate for each sample, including a no-template control. Ct values were averaged for each sample after two PCR experiments. Glyceraldehyde-3-phosphate dehydrogenase (GAPDH) served as the mRNA’s internal standard. The amplification conditions were performed according to the instructions. The primer information is summarized in Supplementary Table 4. Relative quantification of expression was performed compared with the internal standard using the 2 ^-∆∆CT^ method.

### Statistical analysis

The statistical software package SPSS 22.0 (SPSS Inc. Chicago, IL, USA) and Prism 7.0 (GraphPad Software) were used for all statistical analyses. Chi-square analysis was applied to assess differences in ratios among groups. Continuous data are presented as the means ± SD for those that were normally distributed, and the median and quartile ranges for TG that were not normally distributed. Comparison of continuous data sets was performed using Mann-Whitney nonparametric and Kruskal-Wallis tests [36]. R software (version 3.5.0) was used for further bioinformatic analysis. Pheatmap and ggplot2 packages (https://cran.r-project.org/) were utilized for the heat map, volcano plot, PCA analysis and Venn map. To determine whether the methylation level was associated with gene expression, we extracted mRNA data from the genome-wide mRNA expression profile based on same annotated gene symbol with DMaGs, followed by correlation test using GraphPad Prism (Version 7.0) and retesting by SPSS. For those genes with a significant correlation, the mRNA expression levels between CAD patients and healthy controls were compared using the Student’s *t*-test (calculated by GraphPad Prism and retested by SPSS).

## SUPPLEMENTARY MATERIAL

Supplementary Table 1

Supplementary Table 2

Supplementary Table 3

Supplementary Table 4
